# *C9orf72* frontotemporal lobar degeneration is characterised by frequent neuronal sense and antisense RNA foci

**DOI:** 10.1007/s00401-013-1200-z

**Published:** 2013-10-30

**Authors:** Sarah Mizielinska, Tammaryn Lashley, Frances E. Norona, Emma L. Clayton, Charlotte E. Ridler, Pietro Fratta, Adrian M. Isaacs

**Affiliations:** 1 Department of Neurodegenerative Disease, UCL Institute of Neurology, Queen Square, London, WC1N 3BG UK; 2 Department of Molecular Neuroscience, Queen Square Brain Bank, UCL Institute of Neurology, Queen Square, London, WC1N 3BG UK; 3 MRC Centre for Neuromuscular Disease, UCL Institute of Neurology, Queen Square, London, WC1N 3BG UK

**Keywords:** FTLD, FTD, ALS, RNA foci, Antisense, *C9orf72*

## Abstract

An expanded GGGGCC repeat in a non-coding region of the *C9orf72* gene is a common cause of frontotemporal lobar degeneration (FTLD) and amyotrophic lateral sclerosis. Non-coding repeat expansions may cause disease by reducing the expression level of the gene they reside in, by producing toxic aggregates of repeat RNA termed RNA foci, or by producing toxic proteins generated by repeat-associated non-ATG translation. We present the first definitive report of *C9orf72* repeat sense and antisense RNA foci using a series of C9FTLD cases, and neurodegenerative disease and normal controls. A sensitive and specific fluorescence in situ hybridisation protocol was combined with protein immunostaining to show that both sense and antisense foci were frequent, specific to C9FTLD, and present in neurons of the frontal cortex, hippocampus and cerebellum. High-resolution imaging also allowed accurate analyses of foci number and subcellular localisation. RNA foci were most abundant in the frontal cortex, where 51 % of neurons contained foci. RNA foci also occurred in astrocytes, microglia and oligodendrocytes but to a lesser degree than in neurons. RNA foci were observed in both TDP-43- and p62-inclusion bearing neurons, but not at a greater frequency than expected by chance. RNA foci abundance in the frontal cortex showed a significant inverse correlation with age at onset of disease. These data establish that sense and antisense *C9orf72* repeat RNA foci are a consistent and specific feature of C9FTLD, providing new insight into the pathogenesis of C9FTLD.

**Electronic supplementary material:**

The online version of this article (doi:10.1007/s00401-013-1200-z) contains supplementary material, which is available to authorized users.

## Introduction

Frontotemporal lobar degeneration (FTLD) is a common cause of young-onset dementia [15, 24], characterised by atrophy of the frontal and temporal lobes. The three major genetic causes of FTLD are mutations in *MAPT* [16, 23, 27], *GRN* [4, 9], and *C9orf72* [10, 13, 25]. FTLD shares clinical, genetic and pathological features with amyotrophic lateral sclerosis (ALS), and *C9orf72* mutation is the most common known cause of ALS [21]. FTLD has several neuropathological subtypes, the most common of which are cases with tau inclusions (FTLD-tau) and those with TDP-43-positive inclusions (FTLD-TDP) [7, 19]. FTLD-TDP can be further subdivided based on the anatomical and subcellular localisation of the inclusions [18]. Pathologically, *C9orf72* cases fall into two of the four FTLD-TDP subtypes: FTLD-TDP type A, being characterised by many TDP-43-positive neuronal cytoplasmic inclusions and short dystrophic neurites, the majority of which are found in layer 2 of the cortex; or FLTD-TDP type B, characterised by moderate numbers of neuronal cytoplasmic inclusions and dystrophic neurites in all cortical layers [18, 20]. In addition to TDP-43 pathology small neuronal p62 positive ‘star-like inclusions’ are observed predominantly in the hippocampus and cerebellum [2].

The FTD- and ALS-causing mutation in *C9orf72* is an expanded GGGGCC repeat, located upstream of the translation start codon [10, 13, 25]. Expanded repeats in non-coding regions of other genes also cause other diseases with neurodegenerative features, including myotonic dystrophy and certain spinocerebellar ataxias [8]. These diseases are characterised by the aggregation of the expanded repeat RNA into nuclear RNA foci [29]. Intriguingly, it has also been shown that despite being in non-coding regions of the gene, expanded repeats are able to initiate their own translation, a phenomenon termed repeat-associated non-ATG (RAN) translation [30]. The *C9orf72* GGGGCC repeat expansion causes RAN translation, leading to the formation of neuronal inclusions containing RAN protein products generated from sense transcripts [3, 22]. Nuclear RNA foci containing repeats transcribed in the sense direction were reported in post-mortem tissue of four *C9orf72* cases in one study [9], but this was not replicated in another study [26]. *C9orf72* antisense repeat transcripts have been reported [22], but whether antisense transcripts contribute to pathology by generating RNA foci and RAN products, and whether sense RNA foci are a characteristic feature of *C9orf72* FTLD (C9FTLD) are unknown. We report here, in a series of C9FTLD cases and controls, that sense and antisense RNA foci are a characteristic and specific feature of C9FTLD.

## Materials and methods

### Cases

Brain specimens (described in Table 1) were obtained from the Queen Square Brain Bank for Neurological Disorders, UCL Institute of Neurology, London and the MRC London Neurodegenerative Diseases Brain Bank, Institute of Psychiatry, King’s College London. The samples were fixed in 10 % buffered formalin for histopathology and immunohistochemistry. Histological sections from the frontal cortex, hippocampus and cerebellum were analysed. Seven cases had *C9orf72* expansion repeat confirmed by repeat primed PCR (rp-PCR) analysis and Southern blot as previously described [5, 12]. Three cases have been reported previously (cases 11, 12 [20], and case 13, which has a homozygous repeat expansion [12]). Four cases were rp-PCR analysed for this study (cases 14–17). One case (case 18) was determined pathologically using the characteristic p62 pathology found only in *C9orf72* expansion carriers. Additional cases used as controls were three FTLD-TDP type B cases and four FTLD-TDP type A cases without a known genetic mutation (5 of these 7 cases had DNA available and were confirmed by rp-PCR to have normal *C9orf72* repeat length); three Alzheimer’s disease cases all pathologically diagnosed with Braak and Braak stage VI pathology and Thal phase 5 [6, 28]; ten neurologically normal controls. The neuropathological diagnosis was determined using established diagnostic criteria, in line with consensus recommendations for the FTLD spectrum [18]. Immunohistochemistry was performed as previously described [17]. This study was approved by the UCL Institute of Neurology and National Hospital for Neurology and Neurosurgery Local Research Ethics Committee.

**Table 1:** Details of cases used in this study

Case no.	Pathological diagnosis	Gender	Age at onset	Age at death	Duration
1	Normal control	M	NA	85	NA
2	Normal control	F	NA	82	NA
3	Normal control	M	NA	57	NA
4	Normal control	F	NA	79	NA
5	Normal control	F	NA	94	NA
6	Normal control	M	NA	71	NA
7	Normal control	F	NA	68	NA
8	Normal control	F	NA	80	NA
9	Normal control	M	NA	69	NA
10	Normal control	F	NA	93	NA
11	C9FTLD-TDP type A	M	62	72	10
12	C9FTLD-TDP type A	F	66	74	8
13	C9FTLD-TDP type A	M	43	45	2
14	C9FTLD-TDP type B	F	64	66	2
15	C9FTLD-TDP type A	M	53	63	10
16	C9FTLD-TDP type A	M	62	68	6
17	C9FTLD-TDP type A	F	56	67	11
18	C9FTLD-TDP type A	M	54	60	6
19	FTLD-TDP type A	M	57	62	5
20	FTLD-TDP type A	M	75	79	4
21	FTLD-TDP type A	F	75	78	3
22	FTLD-TDP type A	F	83	87	4
23	FTLD-TDP type B	F	75	77	2
24	FTLD-TDP type B	F	63	67	4
25	FTLD-TDP type B	M	67	69	2
26	AD	F	67	76	9
27	AD	M	60	66	6
28	AD	F	51	62	11

### RNA fluorescence in situ hybridisation (FISH) with protein immunostaining

Supplier and catalogue numbers for FISH reagents are detailed in Supplementary Table 1. 7 μm-thick paraffin sections were dewaxed, followed by antigen retrieval in citrate buffer (0.1 M, pH 6) for 20 min in a microwave. Sections were then dehydrated in a graded series of alcohols, air dried and rehydrated in phosphate-buffered saline (PBS), briefly washed in 2×SSC and incubated for 30 min in pre-hybridisation solution (50 % formamide/2×SSC) at 80 °C. Hybridisation solution (50 % formamide, 2×SSC, 0.8 mg/ml tRNA, 0.8 mg/ml salmon sperm DNA, 0.16 % BSA, 8 % dextran sulphate, 1.6 mM ribonucleoside vanadyl complex, 5 mM EDTA, 0.2 ng/μl probe) was heated at 80 °C for 5 min prior to incubation with sections for 2 h at 80 °C. Sections were firstly washed three times for 30 min each with a high-stringency wash solution (50 % formamide/0.5×SSC) at 80 °C, and then three times for 10 min at room temperature in 0.5×SSC. After a brief wash in PBS sections were blocked in 10 % foetal bovine serum/PBS for 30 min, and then incubated with primary antibody (diluted in PBS) overnight at 4 °C, washed with PBS, and then incubated with appropriate secondary antibodies (all Alexa Fluor, Life Technologies at 1:500 in PBS) for 1 h at room temperature. Autofluorescent background was reduced using Sudan black B (0.2 % in 70 % ethanol/PBS, 10 min). After further washes in PBS, sections were mounted with ProlongGold containing DAPI (Life Technologies). Images were obtained using a LSM710 META confocal microscope (Zeiss). For FISH with both probes, the sense probe was applied first as above, and after the three 30 min 80 °C washes in 50 % formamide/0.5×SSC, the antisense probe was added for 2 h, followed by the protocol above. For DNase and RNase treatments the standard protocol above was followed with additional steps: after rehydration in PBS, sections were incubated in 0.5 % Triton/PBS for 10 min, washed once in PBS, and treated with either RNase A (1 mg/ml in PBS/0.05 % Tween-20) or Turbo DNase (Ambion, 200 U/ml in supplied buffer) for 2 h at 37 °C. After the treatment sections were washed three times in PBS, once in 2×SSC and then placed in pre-hybridisation solution and the standard FISH protocol resumed. 2′-*O*-methyl RNA probe (Integrated DNA Technologies) sequences were (GGCCCC)_4_, (GGGGCC)_4_ or (CAG)_7_, 5′ labelled with either Cy3 or Alexa488. Primary antibodies were for NeuN (1:250, Millipore ABN78), GFAP (1:250, Abcam ab4674), Iba1 (1:500, Wako 019-19741), CAII (1:2000, Abcam ab6621), p62 (1:200, Abnova H00008878-M01) and phospho TDP-43 (1:500, Cosmo Bio, CAC-TIP-PTD-P01).

### Quantification of RNA Foci, TDP-43 and p62

Three to six 40×* z*-stack images (45,176 μm^2^ per image) were taken at high resolution (2048 × 2048 pixels) using a 1.4 NA objective, in the anterior frontal cortex, two in the dentate fascia of the hippocampus and two in the granule cell layer of the cerebellum, to ensure a minimum of 100 neurons were quantified in each brain region (approximately 120 neurons in frontal cortex, 350 in hippocampus and 1,200 in cerebellum). Quantification was performed blinded. RNA foci were counted in maximum intensity projections of* z*-stack images using the touch count tool in Volocity image analysis software (Perkin Elmer), and then scored for co-occurrence with staining of the nucleus and cell type markers. RNA foci were determined to be on the edge of the nucleus if they were touching the edge of DAPI staining, and in the cytoplasm if they were outside of the nucleus but within the staining of the cell type markers. Cytoplasmic localisation was not determined for astrocytes or microglia, or neurons of the cerebellum as cytoplasm could not be clearly identified. Cytoplamsic p62 and phospho TDP-43 inclusions were identified by Volocity image analysis software using the same threshold for all images. Inclusions positive for both p62 and phospho TDP-43 were only included in the TDP-43 group, to enable specific quantification of p62-positive, TDP-43-negative inclusions. All data is presented as the mean ± standard error of the mean. The homozygous C9FTLD case is shown on all graphs, but is excluded from means and statistical analysis. Statistical testing was performed with GraphPad Prism software using either a paired *t* test or one-way ANOVA and post hoc Bonferroni test.

## Results

### Neuronal GGGGCC sense RNA foci are a frequent and specific feature of C9FTLD

**Fig. 1:**
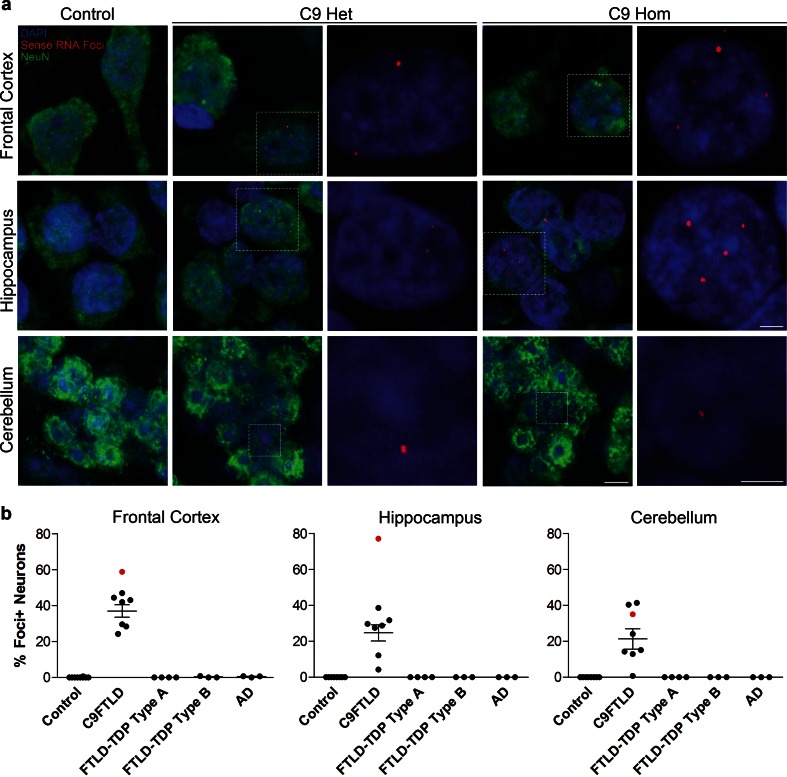
Sense RNA foci are a consistent and specific feature of C9FTLD. **a** RNA FISH for sense foci (*red*) was combined with immunostaining for neurons with NeuN (*green*) and nuclear DNA staining with DAPI (*blue*) in the frontal cortex, hippocampus and cerebellum. Representative images are shown from a neurologically normal control and heterozygous (C9 Het) and homozygous (C9 Hom) *C9orf72* cases. *Boxed* regions are enlarged in the adjacent panel and only *blue* and *red* channels shown. *Scale bar* represents 5 μm in regular panels and 2 μm in enlarged panels. **b** Blind quantification for 10 normal controls, 8 C9FTLD cases, 4 FTLD-TDP type A cases, 3 FTLD-TDP type B cases (all without *C9orf72* mutation) and 3 AD cases reveals sense RNA foci are specific to C9FTLD and present in all brain regions tested. Each *dot* represents an individual case with the homozygous C9FTLD case shown in *red*, and the average and SEM of heterozygous cases shown as *long and short horizontal bars,* respectively

**Fig. 2:**
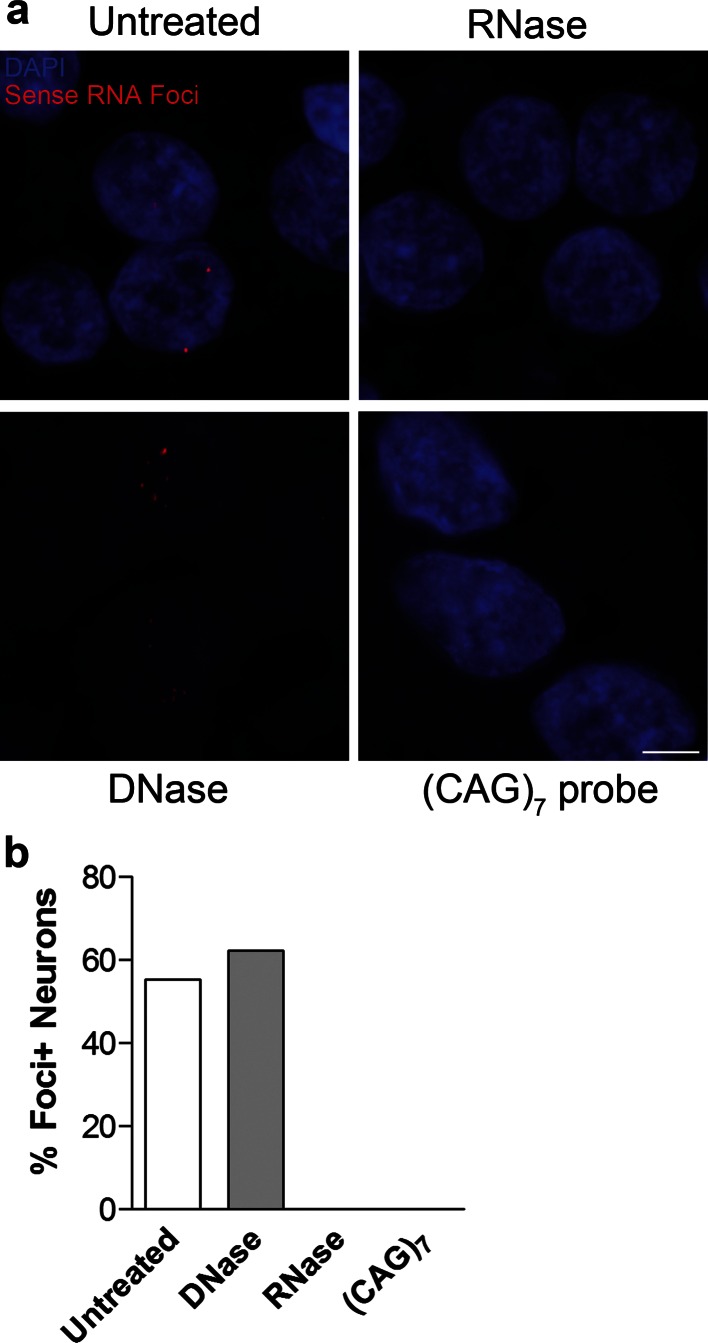
DNase and RNase treatments confirm specificity of RNA foci. **a** RNA FISH for sense foci (*red*) or CTG repeats [(CAG)_7_ probe, *bottom right panel*] was combined with nuclear DNA staining with DAPI (*blue*) in the hippocampus of the homozygous C9FTLD case. RNA foci were not detected after RNase treatment but were still present after DNase treatment, which was confirmed by blind quantification. **b** Loss of DAPI staining (*bottom left panel*) confirmed the DNase was effective. No signal was observed for the (CAG)_7_ probe, suggesting the sense RNA foci are not due to non-specific binding of the RNA probe. *Scale bar* represents 5 μm

To determine whether expanded GGGGCC repeat sense foci are a characteristic and specific feature of C9FTLD, we performed RNA FISH on 8 C9FTLD cases, 7 FTLD-TDP cases without *C9orf72* mutation, 3 Alzheimer’s disease cases and 10 age-matched controls (Table 1). We included a recently described homozygous C9FTLD case that has clinical and pathological features at the severe end of the normal range, including abundant p62-positive inclusions in the frontal and temporal cortices, hippocampus and cerebellum, as well as less abundant TDP-43-positive inclusions [12]. FISH was performed with a fluorescently labelled (GGCCCC)_4_ 2-*O*-methyl RNA probe. In order to improve specificity we utilised the very high melting temperature of the (GGCCCC)_4_ RNA probe (predicted *T*m > 80 °C), using a hybridisation temperature of 80 °C and high-stringency washes. We used high-resolution confocal imaging to improve signal to noise and generated* z*-stacks through the entire volume of the tissue to ensure RNA foci were not missed. We also included an antigen retrieval step, allowing us to combine RNA FISH with protein immunostaining. RNA FISH and immunostaining for the neuronal marker NeuN was performed in the frontal cortex, dentate fascia of the hippocampus and granule cell layer of the cerebellum (Fig. 1a). Quantification (performed blind) revealed GGGGCC sense RNA foci were specific to C9FTLD and occurred in 37 ± 3 % of neurons in the frontal cortex, 25 ± 5 % of granule cell neurons in the hippocampus, and 21 ± 6 % of granule cell neurons in the cerebellum (Fig. 1b). Analysis of individual cases showed the frontal cortex had the highest frequency of sense foci when compared to the other anatomical regions in 6/7 cases (Supplementary Fig. 1). In the homozygous C9FTLD case 59 % of neurons in the frontal cortex contained RNA foci, as did 77 % of granule cells in the hippocampus and 35 % of cerebellar granule cells. To confirm our FISH protocol was detecting RNA and not DNA, we performed FISH on the homozygous C9FTLD case in the presence of either RNase or DNase. As expected, RNase completely removed the RNA foci whereas DNase had no effect (Fig. 2). We also performed FISH with a (CAG)_7_ probe expected to bind expanded repeats in myotonic dystrophy type 1 but not C9FTLD, and again observed no foci in the homozygous C9FTLD case (Fig. 2), further confirming the specificity of our protocol.

### Multiple sense RNA foci can be observed per neuron, and they occur in the nucleus and cytoplasm

**Fig. 3:**
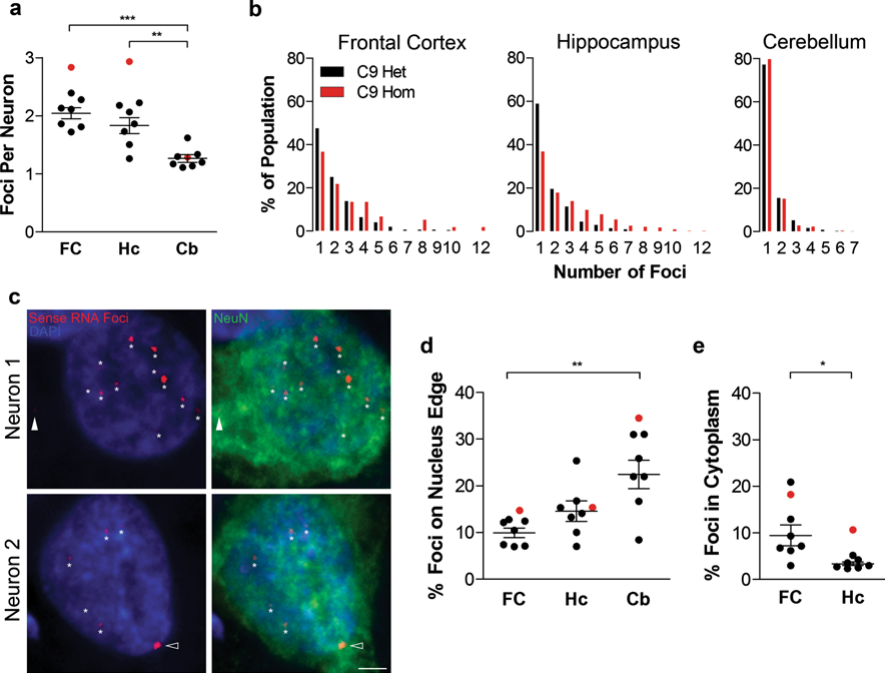
Localisation and frequency of sense RNA foci within neurons. RNA FISH for sense foci (*red*) was combined with immunostaining for neurons with NeuN (*green*) and nuclear DNA staining with DAPI (*blue*) in 8 C9FTLD cases. **a** The number of foci per neuron (in foci containing neurons) was quantified for each case in the frontal cortex (FC) hippocampus (Hc) and cerebellum (Cb). **b** Cumulative frequency distribution of number of foci per neuron in the 7 heterozygous C9FTLD cases (C9 Het, *black bars*) and the homozygous C9FTLD case (C9 Hom, *red bars*). **c** Neuron 1 is a frontal cortical neuron from the homozygous C9FTLD case containing 12 RNA foci (*starred*) including a cytoplasmic foci (*white arrowhead*). Neuron 2 is a frontal cortical neuron from a heterozygous C9FTLD case containing nuclear RNA foci (*starred*) which includes a RNA foci on the edge of the nucleus (*arrowhead*). *Scale bar* represents 2 μm. **d** Quantification of the percentage of neuronal RNA foci that are present on the edge of the nucleus and **e** within the cytoplasm. In **a**, **d** and **e**, each *dot* represents an individual C9FTLD case with the homozygous C9FTLD case shown in *red*, and the average and SEM of heterozygous cases shown as *long and short horizontal bars,* respectively. Significance was determined using one-way ANOVA and post hoc Bonferroni test (**a** and **d**) or paired *t* test (**e**): *p < 0.05, **p < 0.01, ***p < 0.001

We next quantified the number and localisation of sense RNA foci per neuron in the three brain regions. There was an average 2.0 ± 0.1 foci per neuron in the frontal cortex, 1.8 ± 0.1 foci per neuron in the hippocampal dentate fascia, and 1.3 ± 0.1 foci per neuron in the cerebellar granule cell layer (Fig. 3a). Cerebellar granule neurons contained significantly fewer foci per neuron than neurons in the frontal cortex or hippocampal dentate fascia (Fig. 3a). A frequency distribution of the number of RNA foci per neuron showed that in all brain regions neurons most frequently had one focus, and 12 foci was the maximum number observed in a single neuron (Fig. 3b). In the frontal cortex and hippocampus of the homozygous case, neurons contained more foci per cell and had a larger range of foci per cell than in the heterozygous cases (Fig. 3a, b). Neuronal RNA foci were observed predominantly in the nucleus but also in the cytoplasm (for example, neuron 1, Fig. 3c). We also observed foci on the very edge of the nucleus (for example, neuron 2, Fig. 3c). Quantification revealed that significantly fewer neuronal foci were cytoplasmic in hippocampal granule cells (3 ± 0.4 %) than in the frontal cortex (9 ± 2 %, Fig. 3e). 10–22 % of sense RNA foci were present on the edge of the neuronal nucleus in the three brain regions, with cerebellar granule neurons having a significantly higher proportion on the edge of the nucleus than neurons of the frontal cortex (Fig. 3d).

### Sense RNA foci are present in astrocytes, microglia and oligodendrocytes, but at lower frequency than in neurons

**Fig. 4:**
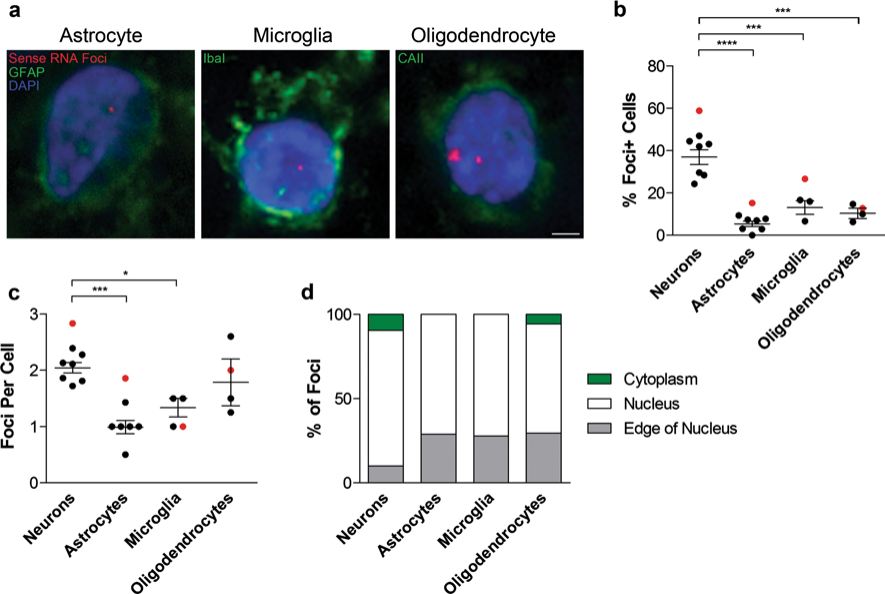
Sense RNA foci are present in the major glial subtypes. **a** RNA FISH for sense foci (*red*) was combined with immunostaining for astrocytes (GFAP), microglia (Iba1) or oligodendrocytes (CAII) (*all in green*), and DAPI (*blue*) in the frontal cortex of 8 C9FTLD cases. *Scale bar* represents 2 μm. The percentage of each cell type containing foci and the number of foci per cell are quantified in (**b**) and (**c**), respectively. **d** Graphical representation of the localisation of sense RNA foci in each cell type. In **b**, **c**, each *dot* represents an individual C9FTLD case with the homozygous C9FTLD case shown in *red*, and the average and SEM of heterozygous cases shown as *long and short horizontal bars,* respectively. Significance was determined using the one-way ANOVA and post hoc Bonferroni test (**b**, **c**): *p < 0.05, ***p < 0.001, ****p < 0.0001

We next performed RNA FISH combined with immunostaining for the astrocyte marker GFAP, the microglial marker Iba1 or the oligodendrocyte marker CAII (Fig. 4a). Quantification in the frontal cortex showed that while sense RNA foci were present in 37 ± 3 % of neurons, they were present in significantly fewer astrocytes (5 ± 1 %), microglia (13 ± 3 %) and oligodendrocytes (10 ± 2 %, Fig. 4b). While oligodendrocytes had a similar number of foci per cell (1.8 ± 0.4) to neurons (2.0 ± 0.1), astrocytes and microglia contained significantly fewer (1.0 ± 0.1 and 1.3 ± 0.2, respectively, Fig. 4c). All three glial subtypes had approximately three times more foci located on the edge of the nucleus than neurons but cytoplasmic RNA foci were only observed in oligodendrocytes (Fig. 4d).

### GGCCCC antisense RNA foci are a frequent and specific feature of C9FTLD

**Fig. 5:**
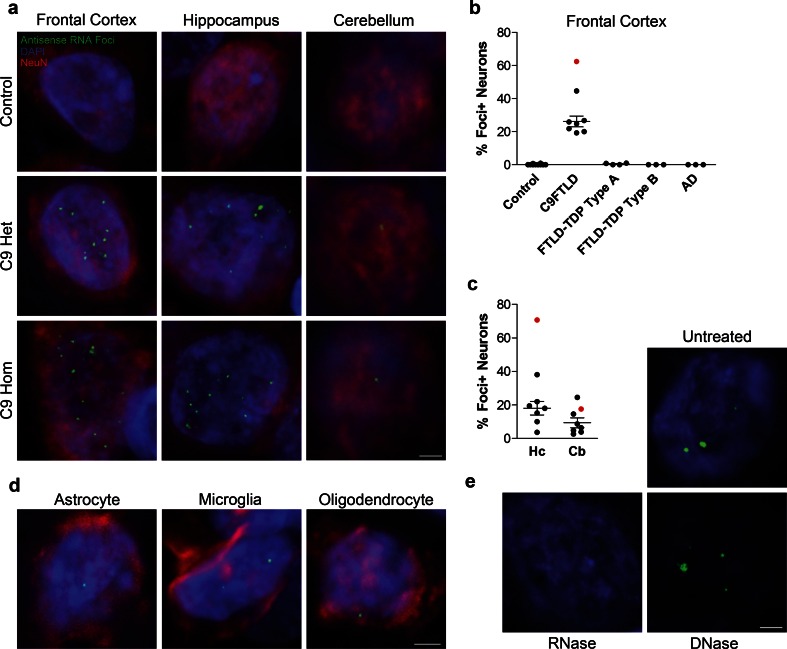
Antisense RNA foci are a consistent and specific feature of C9FTLD. RNA FISH for antisense foci (*green*) was combined with immunostaining for neurons with NeuN (*red*) and nuclear DNA staining with DAPI (*blue*). **a** Representative images from the frontal cortex, hippocampus and cerebellum from a neurologically normal control and heterozygous (C9 Het) and homozygous (C9 Hom) C9FTLD cases. Blind quantification of antisense RNA foci in frontal cortical neurons in C9FTLD cases and controls (**b**) and granule cell neurons of the hippocampus and cerebellum in C9FTLD cases only (**c**). **d** Representative images from a heterozygous C9FTLD case show antisense RNA foci are present in astrocytes, microglia, and oligodendrocytes. **e** RNA FISH for antisense foci was combined with RNase or DNase treatment, confirming the antisense probe detects RNA and not DNA. *Scale bar* represents 2 μm in all panels. In **b**, **c**, each *dot* represents an individual case with the homozygous C9FTLD case shown in *red*, and the average and SEM of heterozygous cases shown as *long and short horizontal bars,* respectively

We then used our optimised combined FISH and protein immunostaining protocol to determine whether antisense RNA foci are also present in C9FTLD. Neuronal antisense RNA foci were present in the frontal cortex, hippocampus and cerebellum of heterozygous and homozygous C9FTLD cases (Fig. 5a). Similarly to sense foci, antisense RNA foci were present in astrocytes, microglia and oligodendrocytes (Fig. 5d). Quantification (performed blind) of antisense foci in the frontal cortex of our series of C9FTLD and control brains showed the antisense foci were specific to C9FTLD and occurred in 26 ± 3 % of frontal cortical neurons (Fig. 5b). Analysis of C9FTLD hippocampus and cerebellum found that 18 ± 4 and 9 ± 3 % of granule cell neurons in these regions contained antisense foci, respectively (Fig. 5c). Across individual cases, the frontal cortex had the highest frequency of antisense foci when compared to the other brain regions in 5/7 cases (Supplementary Fig. 1). DNase and RNase controls also confirmed that we had detected RNA foci and not DNA (Fig. 5e).

### Antisense RNA foci occur in a lower percentage of neurons than sense foci, but the average number of antisense foci per neuron is greater

**Fig. 6:**
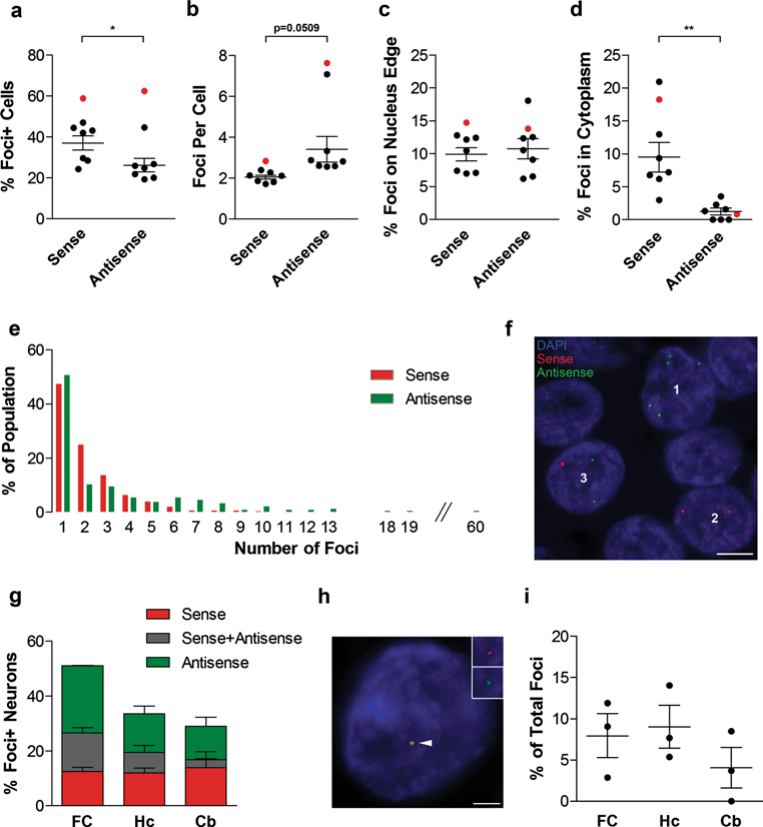
Comparison of sense and antisense RNA foci. **a–e** RNA FISH for either sense foci or antisense foci was combined with NeuN and DAPI staining in the frontal cortex of 8 C9FTLD cases. The percentage of neurons containing each foci type (**a**), the number of foci per cell (**b**), and their locations (**c**) and (**d**) were compared. **e** Frequency distribution of number of sense foci (*red bars*) and antisense foci (*green bars*) per neuron in the 7 heterozygous C9FTLD cases. The maximum number of sense RNA foci in a single neuron was 10 compared with 60 for antisense foci. **f–i** Sequential labelling of both sense and antisense foci was combined with NeuN and DAPI staining in a subset of C9FTLD cases in the frontal cortex (FC), hippocampus (Hc) and cerebellum (Cb). **f** Representative image from the hippocampus of the homozygous C9FTLD case showing neurons containing exclusively antisense foci (neuron 1), exclusively sense foci (neuron 2) or both antisense and sense foci (neuron 3). *Scale bar* represents 5 μm. **g** Quantification of the percentage of neurons containing sense foci, antisense foci, or both. **h** A neuron from the hippocampus of a heterozygous C9FTLD case showing co-localisation of sense and antisense foci (*arrowed*). *Insets* show enlarged region containing *arrowed* foci with individual channels for foci type and DAPI. *Scale bar* represents 2 μm. **i** Quantification of sense and antisense co-localisation from 3 heterozygous C9FTLD cases in all 3 brain regions. In **a**–**d** and **i**, each *dot* represents an individual C9FTLD case with the homozygous C9FTLD case shown in *red*, and the average and SEM of heterozygous cases shown as *long and short horizontal bars,* respectively. Significance was determined using a paired *t* test: *p < 0.05, **p < 0.01

We next compared the frequency and location of sense and antisense RNA foci. Antisense RNA foci occurred in significantly fewer frontal cortical neurons than sense foci (26 vs. 37 %, Fig. 6a). However, the average number of antisense RNA foci per neuron was significantly greater than for sense foci (3.4 ± 0.6 vs. 2.0 ± 0.1, Fig. 6b). Similar but non-significant trends were observed in hippocampal and cerebellar granule neurons, with a lower percentage of cells containing antisense than sense foci (18 vs. 25 %, and 9 vs. 21 %, Figs. 1b, 5c), and more antisense than sense foci per neuron in the hippocampus (2.6 vs. 1.8, Fig. 3a; Supplemental Fig. 2), but not in the cerebellum (Fig. 3a; Supplemental Fig. 2). The maximum number of foci observed in a single neuron was also much higher for antisense foci than sense foci (60 vs. 10 in heterozygous cases, Fig. 6e). In the frontal cortex of the homozygous case, the percentage of neurons containing sense or antisense foci was similar (59 vs. 62 %), and the number of antisense foci per neuron was much higher than for sense foci (7.6 vs. 2.8).

In the frontal cortex of the heterozygous C9FTLD cases the percentage of sense and antisense foci that occurred on the edge of the nucleus was very similar (10 vs. 11 %, Fig. 6c). The percentage of foci located in the cytoplasm, however, was significantly lower for antisense than sense foci (1 vs. 9 %, Fig. 6d). No differences in the localisation of antisense foci as compared to sense foci were seen in other brain regions (Fig. 3d, e; Supplementary Fig. 2). The localisation of antisense foci in the homozygous case was similar to the heterozygous cases (Fig. 6; Supplementary Fig. 2).

We performed sequential FISH with the sense probe followed by the antisense probe to determine whether sense and antisense foci occur in the same neurons. We observed sense and antisense foci in distinct neurons (neurons 1 and 2, Fig. 6f), distinct sense and antisense foci co-occurring within the same neuron (neuron 3, Fig. 6f), and co-localisation of sense and antisense foci (Fig. 6h). Quantification revealed that sense and antisense foci co-occur in 14 ± 3, 7 ± 5 and 3 ± 0.5 % of neurons in the frontal cortex, hippocampus and cerebellum, respectively (Fig. 6g). This is approximately the co-occurrence that would be predicted based on relative frequencies. We quantified the co-localisation of sense and antisense foci and found that <15 % of foci co-localised in all brain regions (Fig. 6i). This analysis also reveals total foci (sense and antisense) burden in each brain region. 51 ± 6 % of neurons in the frontal cortex were found to contain foci, as did 34 ± 6 and 29 ± 6 % of granule cell neurons in the hippocampus and cerebellum, respectively (Fig. 6g). This is an underestimate of foci burden, as approximately a 10 % reduction in sense foci was noted using the double staining protocol, probably due to decreased sensitivity caused by the additional time at 80 °C during antisense probe hybridisation and washes.

### Sense and antisense RNA foci occur in neurons containing p62- or TDP-43-positive inclusions

**Fig. 7:**
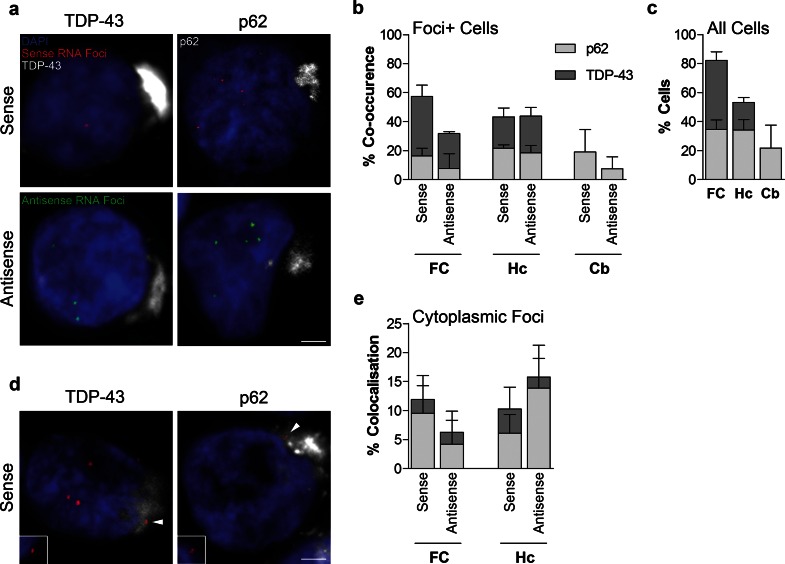
Comparison of RNA foci, p62 and TDP-43 inclusions in C9FTLD patient brain. RNA FISH for either sense or antisense foci was combined with immunostaining for p62 or phospho TDP-43 in the frontal cortex (FC), hippocampus (Hc) and cerebellum (Cb) of 3 heterozygous C9FTLD cases. **a** Representative images from the frontal cortex show that both sense and antisense foci occur in the same neurons as either p62-positive (TDP-43 negative) or TDP-43 inclusion pathology (*both shown in white*). **b** Quantification of co-occurrence of sense or antisense foci with p62 or TDP-43 pathology within the same cell in the frontal cortex, hippocampus and cerebellum shows no obvious associations between pathologies when compared to relative frequencies of each inclusion pathology in the total cell population in respective brain regions (**c**). **d** Representative images show that cytoplasmic sense foci (*arrowed*) can occasionally be found to co-localise within both p62 and TDP-43 inclusions in the hippocampus. *Insets* show enlarged region containing *arrowed* foci for each image without channel for p62 or TDP-43 to show clearly that foci are outside the nucleus. **e** Quantification of the co-localisation of cytoplasmic sense or antisense foci with either p62 or TDP-43 inclusions in the frontal cortex and hippocampus. *Scale bars* represent 2 μm. Data in graphs is shown as the average and SEM

We next investigated the relationship of RNA foci to TDP-43 and p62 inclusions. Both sense and antisense foci containing cells were found to also contain p62-positive (TDP-43-negative) or TDP-43-positive cytoplasmic inclusions (Fig. 7a). Quantification showed that the frequencies of sense or antisense foci containing cells also containing p62- or TDP-43-positive inclusions (Fig. 7b), when compared to the relative frequencies of p62 and TDP-43 inclusions in the same brain region (Fig. 7c), showed no clear association between pathologies in any brain region. Cytoplasmic sense or antisense foci that co-localised with p62- or TDP-43-positive inclusions were identified (Fig. 7d), but quantification revealed that this only occurred for a minority (<15 %) of the cytoplasmic foci (Fig. 7e).

### Foci burden correlates with age at onset of C9FTLD

**Fig. 8:**
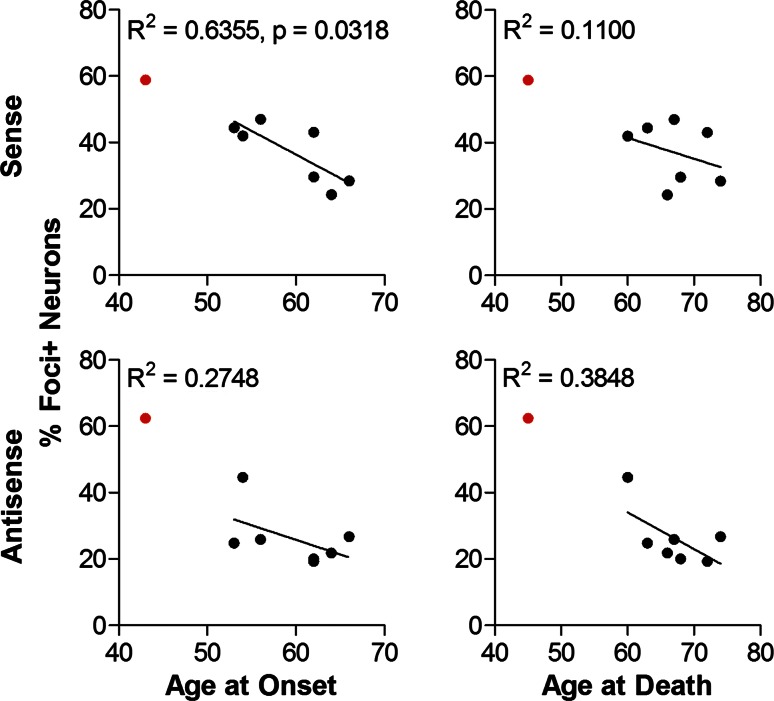
Clinical phenotypes correlate with the percentage of foci containing neurons in the frontal cortex of C9FTLD patient brain. Age at onset of disease or age at death was plotted against percentage of sense or antisense foci containing neurons in the frontal cortex for each individual case. Linear regressions were performed on data from heterozygous C9FTLD cases, with *R* ^2^ and any significant *p* value shown as a measure of goodness-of-fit and significance of the linear trend. Each *dot* represents an individual C9FTLD case with the homozygous C9FTLD case shown in *red*, even though it is not included in the linear regression analysis

To gain further insights into pathomechanisms of C9FTLD, sense and antisense neuronal RNA foci burden (percentage of neurons containing foci) were correlated with age at onset and age at death. Sense foci burden in the frontal cortex was found to be significantly and inversely correlated with age at onset of disease (Fig. 8). A consistent trend for inverse correlation was observed for sense foci burden with both age at onset and age at death in the frontal cortex, hippocampus and cerebellum (Fig. 8; Supplementary Fig. 3). Antisense foci burden only showed a trend for inverse correlation in the frontal cortex (Fig. 8; Supplementary Fig. 3). In summary, these data suggest that both sense and antisense foci are important pathologies in C9FTLD.

## Discussion

We provide the first definitive evidence for the presence of both sense and antisense RNA foci in C9FTLD. This has important implications for disease mechanism. Firstly, it suggests that either or both sense and antisense RNA foci could play a pathogenic role in C9FTLD; presumably through sequestration of RNA-binding proteins, as has been observed in other non-coding repeat expansion diseases [8]. The identities of the sequestered proteins in patient RNA foci are currently unknown. Our detection of antisense as well as sense RNA foci means identification of proteins sequestered by antisense transcripts will be of great interest. We observed that fewer neurons contained antisense than sense foci, but that there were a greater number of antisense RNA foci per neuron. However, it appeared that the antisense foci were generally smaller than the sense foci, so it is unclear whether one type of foci has the potential to sequester more protein than the other. We have previously shown that the sense (GGGGCC)n RNA forms a G-quadruplex structure [11], which could mediate sequestration of proteins. Cytosine-rich sequences can form a distinct four-stranded structure termed an i-motif [14]. It will be important to determine whether the (GGCCCC)*n* sequence forms an i-motif and whether this has biological significance. The identification of cytoplasmic antisense RNA foci raises the possibility that antisense RAN translation products will be produced, as well as the sense RAN translation products already reported [3, 22], further increasing the potential pathogenic species in C9FTLD.

Sense RNA foci were initially reported in 25 % of cells in the frontal cortex of 2 C9FTLD cases and the spinal cord of 2 C9ALS cases [10], but a second study was unable to replicate these findings and observed RNA foci in controls [26]. Sense RNA foci have also been detected in C9FTLD-derived induced pluripotent stem cells [1]. We report the first RNA FISH study on a series of C9FTLD cases, and both normal and neurodegenerative disease controls, and show that using our optimised FISH protocol, both GGGGCC sense and GGCCCC antisense RNA foci are specific to C9FTLD. We observed sense and antisense foci in 37 and 26 % of frontal cortical neurons, respectively, and a total frontal cortical neuronal foci burden (sense and antisense) of 51 %, which shows that foci are a major pathology in C9FTLD. We used a much higher hybridisation temperature than the previous studies (possible due to the extremely high melting temperature of our 2′-*O*-methyl RNA probes), and stringent washes, which might explain the greater specificity. We also note that the RNA foci were not visible using a standard fluorescence microscope, but were readily visible with a confocal microscope, which may also explain differences with previous studies. It is likely that the enhanced signal to noise provided by high-resolution microscopy enabled sensitive detection of the RNA foci, which are, therefore, likely to be small structures. Our use of* z*-stacks to image the entire volume of the cell allowed a more accurate estimation of RNA foci per cell and the percentage of neurons containing foci than using confocal images from a single plane, which, given the small size of foci, would lead to an underestimation of foci numbers.

We identified sense and antisense RNA foci in astrocytes, microglia and oligodendrocytes, but at lower frequency than in neurons. This suggests neurons are the primary sites of foci pathology but also raises the possibility of impaired glial function due to RNA foci formation. It will be interesting to determine whether the same repertoires of RNA-binding proteins are sequestered in these different cell types, and to understand why neurons have a greater RNA foci burden. Although foci were present in all three brain regions, the highest burden of foci was consistently found in the frontal cortex, which is the region that suffers the greatest neuronal loss in FTLD. A previous study on the homozygous case described the clinical and pathological features as severe, but not completely outside of the usual disease spectrum [12]. Our data show that the homozygous case displays severe sense and antisense RNA foci pathology when compared to the heterozygous cases, with a higher proportion of neurons containing foci and also more foci per cell. This difference was most striking in the frontal cortex and hippocampus, with no apparent difference in the cerebellum. The severe foci burden and early age at onset of the homozygous case are consistent with our observation that foci burden inversely correlates with age at onset. However, it will be important to replicate this finding in a larger series.

The majority of RNA foci in all cell types were located in the nucleus, consistent with a role in nuclear RNA-binding protein sequestration. In the different brain regions between 10 and 22 % of neuronal foci were located at the very edge of the nucleus, with the cerebellum having a significantly higher proportion than the frontal cortex. The significance of foci on the edge of the nucleus is unclear but it would be interesting to determine whether they are in proximity to nuclear pores and whether they have the potential to block nuclear RNA import or export. 9 % of sense RNA foci in the frontal cortex were cytoplasmic, consistent with RAN translation of the repeat transcripts, which would require the cytoplasmic translation machinery. Significantly fewer cytoplasmic antisense foci were found in the frontal cortex, which may reflect differential nuclear export or turnover. Neither sense nor antisense foci showed an obvious association with p62- or TDP-43-positive inclusions, although cytoplasmic foci were occasionally found within both types of inclusion in all brain regions where they are found. This suggests that the presence of RNA foci does not predict the presence of TDP-43- or p62-positive inclusions. Co-localisation analyses with antibodies specific for each RAN product will be required to determine whether individual sense or antisense RAN products are associated specifically with sense or antisense RNA foci.

Our identification of sense and antisense RNA foci, and the significant correlation between sense foci burden and age at onset, add weight to the possibility that toxic RNA gain of function is involved in C9FTLD pathogenesis, but does not rule out a role for loss of *C9orf72* function or RAN translation products. The identification of proteins sequestered by RNA foci will be a key step in confirming the validity of the toxic RNA hypothesis. In conclusion, we have identified both GGGGCC sense and GGCCCC antisense RNA foci as a consistent and specific feature of C9FTLD in a series of cases and controls. These findings have important implications for understanding disease mechanism and may help inform the drug discovery process.

## Electronic supplementary material

Below is the link to the electronic supplementary material.

Supplementary material 1 (PDF 685 kb)
