# Supplementary material for: C9orf72 frontotemporal lobar degeneration is characterised by frequent neuronal sense and antisense RNA foci
Source: Acta Neuropathol. 2013 Oct 30;126(6):845–57. doi: 10.1007/s00401-013-1200-z (PMC3830745; doi:10.1007/s00401-013-1200-z)
Supplement: Supplementary file 1 — Supplementary material 1 (PDF 685 kb) [file 401_2013_1200_MOESM1_ESM.pdf]

C9orf72 frontotemporal lobar degeneration is characterised by frequent neuronal sense and antisense RNA foci

Acta Neuropathologica

Sarah Mizielinska, Tammaryn Lashley, Frances Norona, Emma L. Clayton, Charlotte E. Ridler, Pietro Fratta and Adrian M. Isaacs.

Corresponding author:

Dr Adrian Isaacs

Department of Neurodegenerative Disease, UCL Institute of Neurology, Queen Square, London WC1N 3BG

a.isaacs@prion.ucl.ac.uk

Supplementary Table 1. Supplier and catalogue numbers for FISH reagents used in this study.

Reagent	Supplier	Catalogue number
Formamide deionized	Amresco	606
20x SSC Buffer	Sigma	S6639
tRNA from baker's yeast	Sigma	R5636
ssDNA from salmon testes	Sigma	D7656
BSA	Sigma	B8667
Dextran sulphate	Sigma	D8906
EDTA	Sigma	E5134
Ribonucleoside vanadyl complex	New England Biolabs	S1402S
RNase A	Sigma	R6513
TURBO™ Dnase	Ambion	AM2238

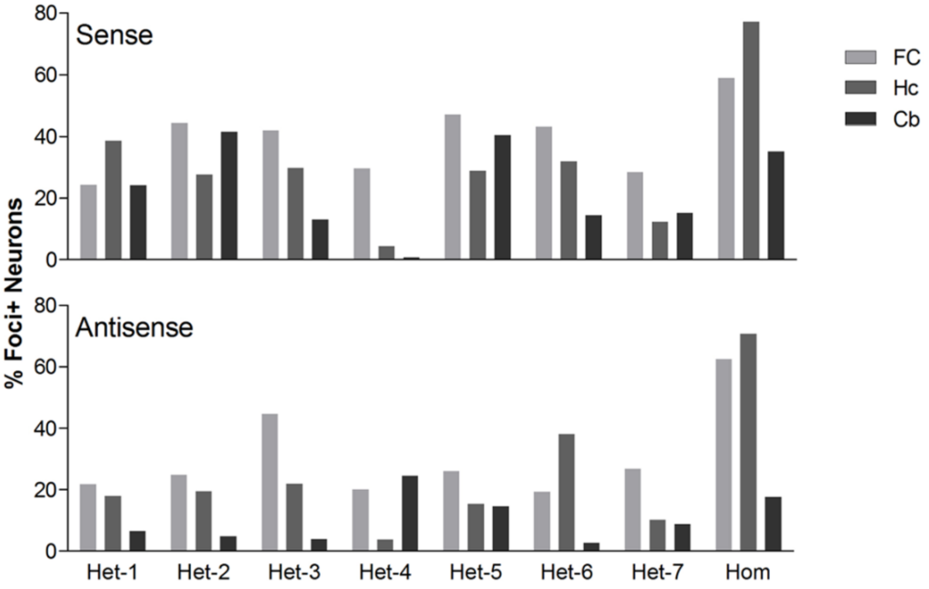

Supplementary Fig. 1 Differences in anatomical distribution of sense and antisense foci are relatively consistent between cases. Bar graphs show percentage of sense or antisense foci-containing neurons in the frontal cortex (FC), hippocampus (Hc) and cerebellum (Cb) for individual heterozygous C9FTLD cases (Het 1-7) and the homozygous C9FTLD case (Hom).

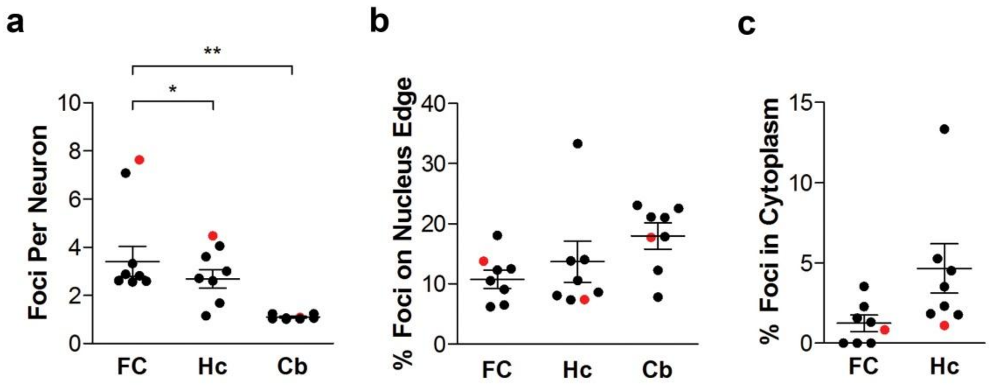

Supplementary Fig. 2 Localisation and frequency of antisense RNA foci within neurons. RNA FISH for sense foci was combined with immunostaining for neurons with NeuN and nuclear DNA staining with DAPI in the frontal cortex (FC), hippocampus (Hc) and cerebellum (Cb) of 8 C9FTLD cases, as shown in Figure 5. **(a)** The number of foci per neuron was quantified for each case. Significantly more antisense RNA foci per neuron were found in the frontal cortex and hippocampus than in the cerebellum, as was found for sense foci. **(b)** Quantification of the percentage of neuronal antisense RNA foci that are present on the edge of the nucleus and **(c)** within the cytoplasm. In each panel, each dot represents an individual C9FTLD case with the homozygous C9FTLD case shown in red and the average and SEM of heterozygous cases shown as long and short horizontal bars respectively. Significance was determined using one-way ANOVA and *post hoc* Bonferroni test: *$p<0.05$, **$p<0.01$.

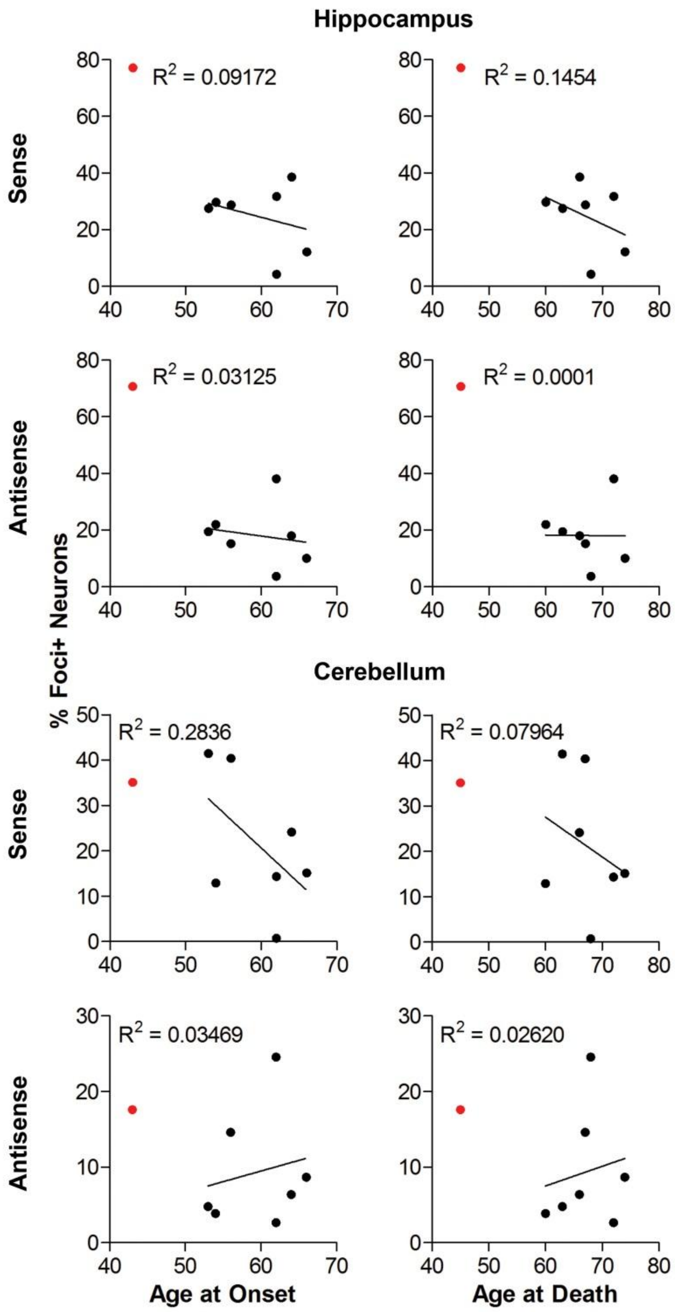

Supplementary Fig. 3 Clinical phenotype correlation with neuronal foci burden in the hippocampus and cerebellum of C9FTLD patient brain. Age at onset of disease or age at death were plotted against percentage of sense or antisense foci-containing neurons in the hippocampus and cerebellum for each individual case. Linear regressions were performed on data from heterozygous C9FTLD cases, with R^2 shown as a measure of goodness-of-fit of the linear trend. Each dot represents an individual C9FTLD case with the homozygous C9FTLD case shown in red, even though it is not included in the linear regression analysis.
